# Supplementary figures and images for: The impact of Connexin 43 deficiency on the cell shape and cytoskeleton of murine Sertoli cells: A house with ramshackle walls?
Source: PLoS One. 2025 Apr 24;20(4):e0321292. doi: 10.1371/journal.pone.0321292 (PMC12021162; doi:10.1371/journal.pone.0321292)

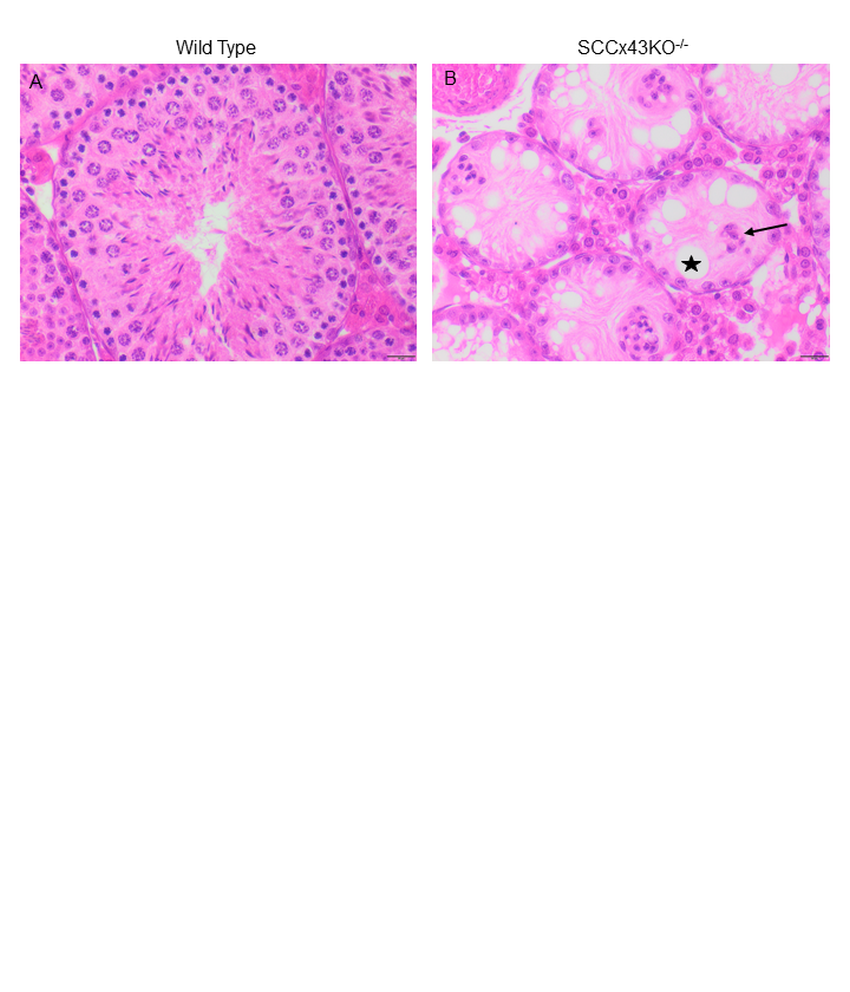

Supplement: S1 Fig — Wild type mice feature seminiferous tubules with normal spermatogenesis. However, Connexin 43 deficient tubules exhibit an arrest of spermatogenesis at level of spermatogonia or Sertoli cell-only syndrome (SCOS). Furthermore, in SCCx43KO-/--mice, the seminiferous tubules are highly vacuolated (star) and feature intratubular cell cluster (arrow); scale bars = 20 µm. (TIF) [file pone.0321292.s001.tif]

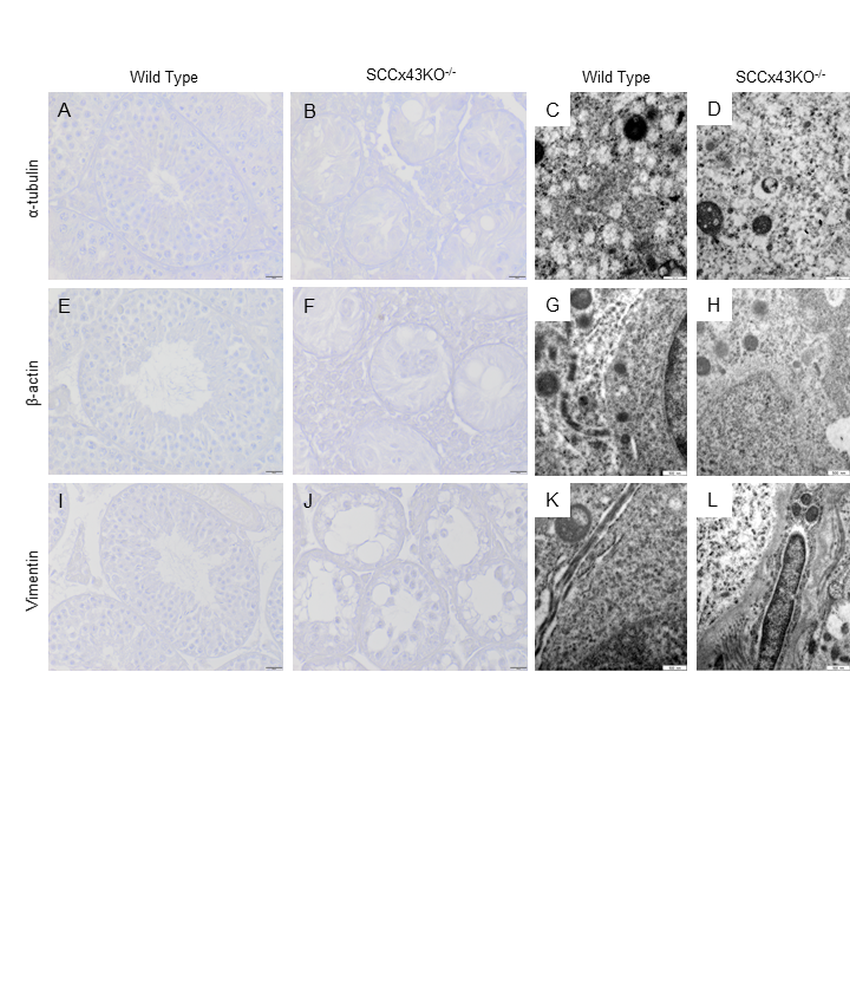

Supplement: S2 Fig — Scale bars = 20 µm (A, B, E, F, I, and J), 500 nm (C, D, G, H, K, and L). (TIF) [file pone.0321292.s002.tif]

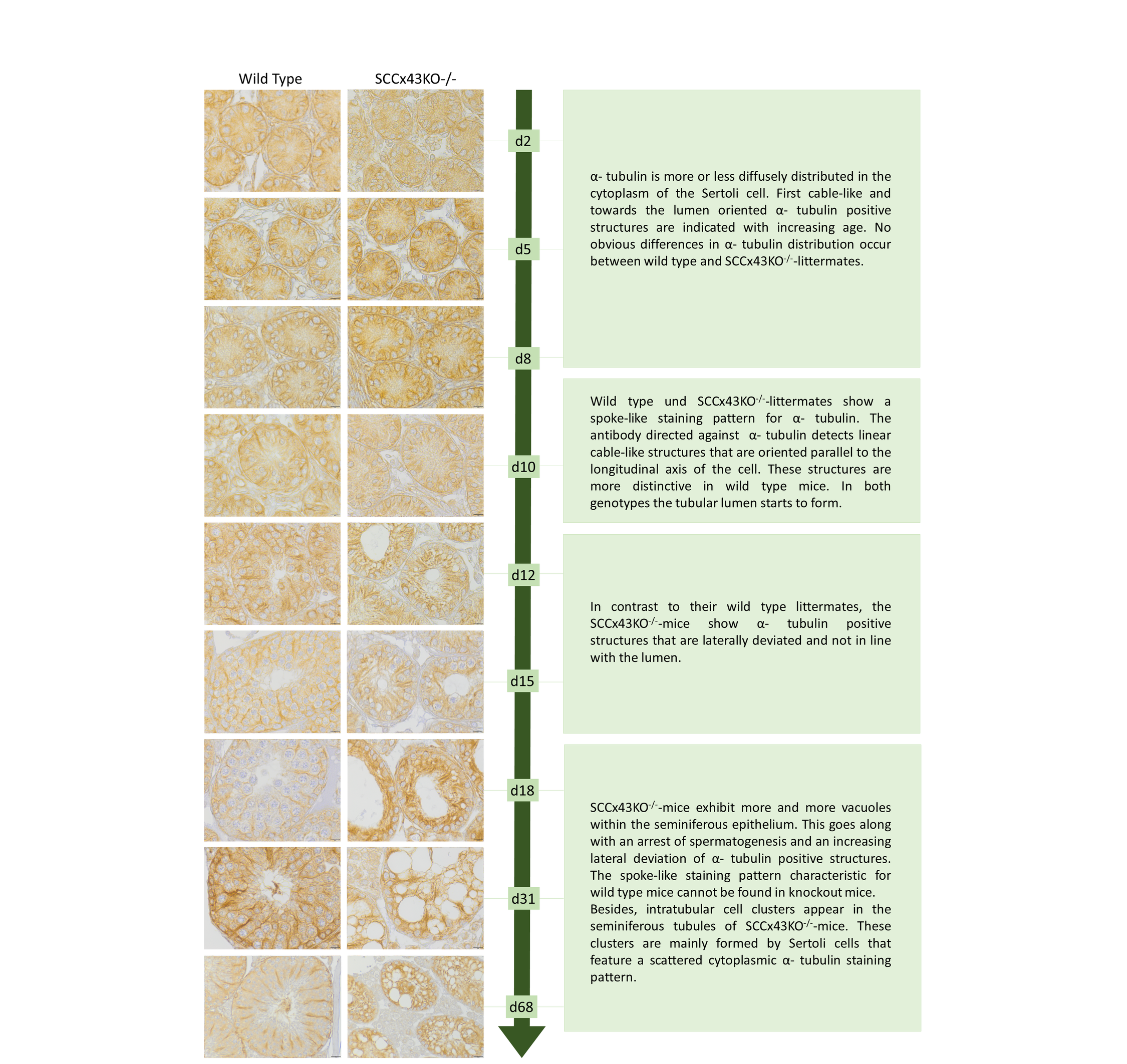

Supplement: S3 Fig — (TIF) [file pone.0321292.s003.tif]

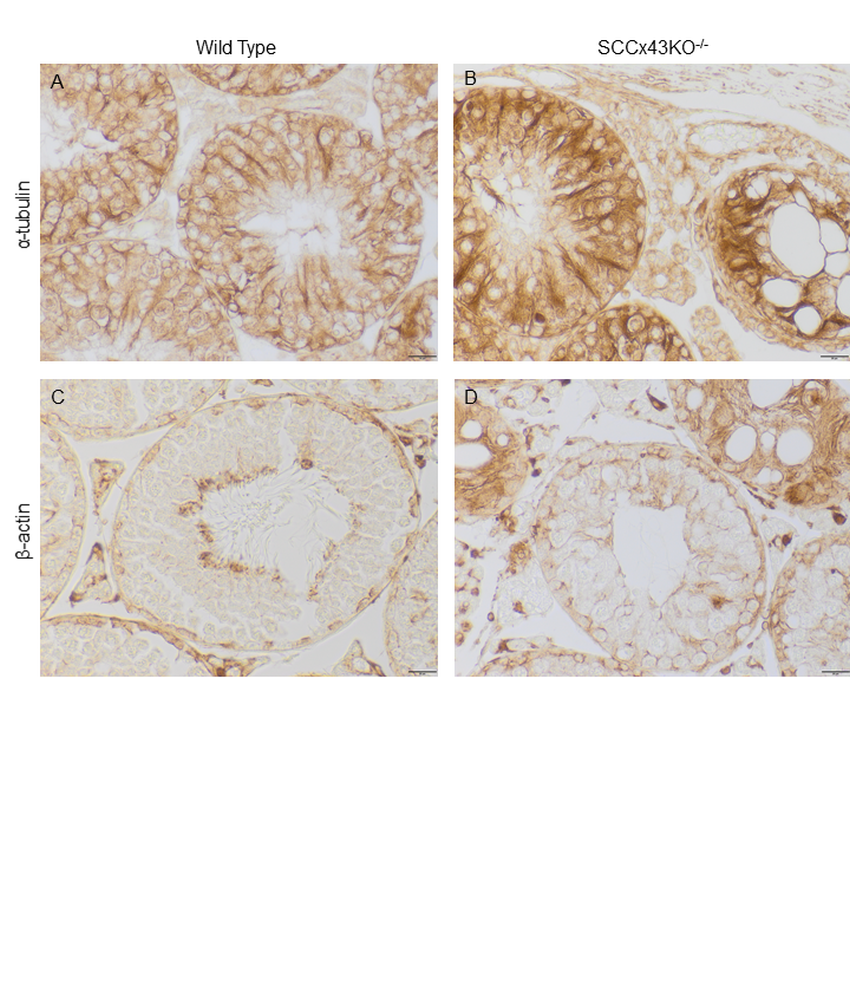

Supplement: S4 Fig — The antibody against α-tubulin causes a radial staining pattern in wildtype mice and in those tubules of SCCx43KO-/--mice which feature residual spermatogenesis. Immunolabeling for β-tubulin in wild type mice can be found near the basement membrane just above the spermatogonia as well as around elongating spermatids. While most Connexin 43 deficient tubules immunostained diffusely positive for β-actin, the tubules with residual spermatogenesis show an analogical “normal” staining pattern; scale bars = 20 μm. (TIF) [file pone.0321292.s004.tif]

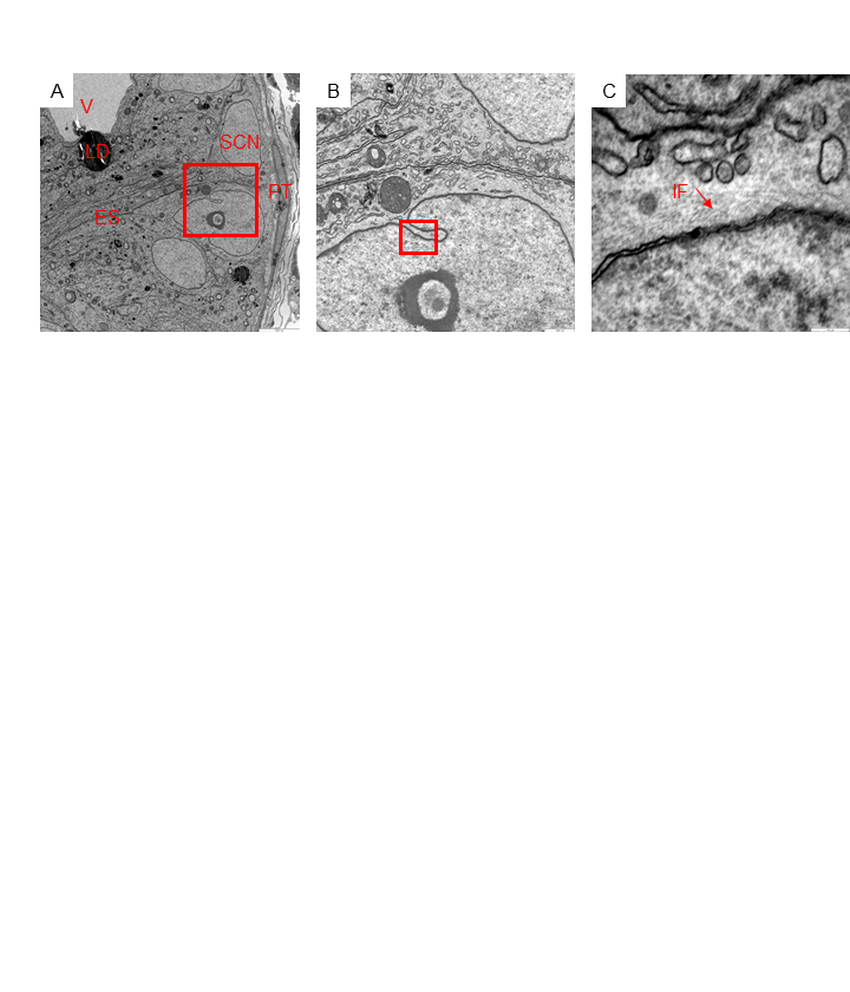

Supplement: S5 Fig — The red boxes are shown in higher magnification in B and C. Intermediate filaments (IF) surround the Sertoli cell nucleus (SCN); scale bars = 5000 nm (A), 1000 nm (B), 250 nm (C). (TIF) [file pone.0321292.s005.tif]
