# Supplementary material for: The impact of Connexin 43 deficiency on the cell shape and cytoskeleton of murine Sertoli cells: A house with ramshackle walls?
Source: PLoS One. 2025 Apr 24;20(4):e0321292. doi: 10.1371/journal.pone.0321292 (PMC12021162; doi:10.1371/journal.pone.0321292)
Supplement: Attachment — Submitted filename: Response to Reviewers.doc [file pone.0321292.s013.doc]

To the editor

PLOS ONE

_Anatomisches Institut, Stiftung Tierärztlich Hochschule Hannover_	Anatomisches Institut

^Bischofsholer Damm 15, 30173 Hannover^	Stiftung Tierärztliche Hochschule Hannover

**Dr. Sarah Gniesmer**

Bischofsholer Damm 15

30173 Hannover

Tel.: (05 11) 8 56-77 11

Fax: (05 11) 8 56-76 83

Email: sarah.gniesmer@tiho-hannover.de

Hannover, 05/02/2025

Dear ladies and gentleman, dear editor,

Please find enclosed our revised version of the manuscript. We have amended and rewritten the manuscript according to the reviewers' comments and hope that this revised version is now suitable for publication in PLOS ONE.

I would like to thank the two reviewers for taking the time to improve our manuscript with their comments. We hope that they will now be pleased with the changes made and approve the publication.

In response to comments No.1 and 2 (Reviewer 1) we performed different co-staining experiments (e.g. Connexin 43 and alpha-tubulin; Connexin 43 and beta-galactosidase; Sox 9 and Claudin 11). Representative figures can be found below in this cover letter. These new data might be added to the final version of the manuscript depending on the reviewers and/or editors decision.

As suggested, I would now like to respond to you and the reviewers, point by point, to the comments and advice below.

We look forward to hearing from you, and if you should have any further questions, please do not hesitate, and contact us.

On behalf of all other authors,

Sincerely,

Sarah Gniesmer

**Journal Requirements:**

1. The manuscript has been checked for Plos One style requirements.
2. A part of the manuscript was published as a doctoral thesis in June 2023 as mentioned in the first cover letter. This doctoral thesis is a German-language publication (except for an English summary), which is only available via the publication server “Tiho elib” (repository of the University of Veterinary Medicine Hannover Foundation). In order to make the data available to a broader, international readership, we would like to publish it in the form of the submitted manuscript.
3. As the data is not one of the core topics of the manuscript, we have decided to remove this sentence from the manuscript. This change is marked accordingly in the file “Revised manuscript with Track Changes”.
4. The captions of the supporting information have now been inserted at the end of the manuscript (after the references).
5. The reference list was checked for completeness and correctness and no errors were found.

**Comments of the Reviewers:**

Reviewer 1:

First of all, we would like to thank this reviewer/you for your praise of our manuscript and especially for your comments.

Figure 3 implies differences in alpha-tubulin organization between wild type and SCCx43KO-mice. The very good, justified and reasonable question of this reviewer is whether either the gap junction protein Connexin 43 (Cx43) helps in acting as an anchor for the observed radial spike like organization of microtubules or whether the altered distribution pattern of alpha-tubulin in Cx43-deficient Sertoli cells is caused by the lack of (differentiated) germ cells.

We also thank this reviewer for the idea to perform co-staining experiments of alpha-tubulin and Cx43 in wild type mice.

Results (and data from the literature) do favour the hypothesis that germ cells are more likely involved in microtubule organization and distribution (rather than the gap junction protein Cx43 itself) as there seems to be no direct correlation between Cx43-synthesis and alpha-tubulin immunolocalization.

In wild type mice, using co-staining against Cx43 (red) and alpha-tubulin (brown), we detected

1. seminiferous tubules / Sertoli cells that are immunopositive for Cx43 (Cx43+) and immunopositive for alpha-tubulin (α tubulin +, with the typical spike like staining pattern) (Fig. A * and Fig. D *; LC = Leydig cells, scale bar = 20 µm)

2. seminiferous tubules / Sertoli cells that are immunopositive for Cx43 (Cx43 +) and/but no spike like staining pattern for alpha-tubulin (Fig. B ■; LC = Leydig cells, scale bar = 20 µm) and

3. seminiferous tubules / Sertoli cells that are immunonegative for Cx43 (Cx43 -) and no spike like staining pattern for alpha-tubulin (Fig. A ▲; scale bar = 20 µm)

4. seminiferous tubules / Sertoli cells that are immunonegative for Cx43 (Cx43 -) and immunopositive for alpha-tubulin (α tubulin +) (Fig. C ●, Fig. D ●; LC = Leydig cells, scale bar = 20 µm)

In addition it has to be kept in mind that there exists a stage dependent synthesis of Cx43 in adult wild type mice and rats (see e.g. RISLEY et al. 1992, BATIAS et al. 1999) making the interpretation (link between Cx43 and tubulin) of the results in wild type mice even more complex. In mature murine testes, Cx43 immunoreactivity on Sertoli cell surfaces is strong in stages VI-VIII and consistently weak or negative in stages IX-XII (RISLEY et al. 1992).

As each stage in a cycle of the seminiferous epithelium is defined by the association of specific germ cell stages in discrete patterns along the length of the seminiferous tubule, microtubule organization might more likely be regulated or influenced by specific germ cells. This idea can be supported by the results obtained from our SCCx43KO-model.

In most seminiferous tubules of adult SCCx43KO-mice, no differentiated germ cells develop/arise and the alpha-tubulin distribution pattern is altered.

1. Seminiferous tubules showing an arrest of spermatogenesis at the level of spermatogonia or even and SCO-syndrome:

No connexin43 in Sertoli cells = no differentiated germ cells = altered distribution of alpha tubulin

2. Seminiferous tubules showing residual spermatogenesis (up to elongated spermatids):

No connexin43 in Sertoli cells but differentiated germ cells = normal distribution pattern of alpha tubulin

What implies the literature:

Also in rat seminiferous tubules there exists a stage-specific alpha-tubulin staining pattern in Sertoli cells (WENZ and HESS 1998) supposing a regulating role of (stage dependent) germ cells on alpha-tubulin distribution.

In testes from adult (140-day-old) SCARKO-mice (with an arrest in meiosis), Sertoli cells showed a weaker immunoreactivity and a more variable staining pattern for beta-tubulin. Some tubules showed typical spoke-like structures comparable to those observed in control testes, whereas others displayed a more irregular „honeycomb“ pattern of beta-tubulin staining (DE GENDT et al. 2011).

In another experimental study (using w/w^wei^ mutant mice that are devoid of germ cells) it was shown the absence of germ cells apparently affects also the microtubular cytoskeleton (WOLF and WINKING 1996).

And finally, results from the present study (Figure 3, E) show that first/initially with increasing age (here: d15 post partum), wild type mice develop the characteristic spoke-like staining pattern of alpha-tubulin concomitant with the appearance of further differentiated germ cell populations (see also Supporting figure S 3 Fig.: there: d10 post partum onwards).

Some additional comments:

Probably the most important component of the cytoskeleton for maintaining a Sertoli cell’s shape are the microtubules (RUSSELL et al. 1981; AMLANI and VOGL 1988). Various studies using substances that bind to microtubules have also shown that the microtubules are important for the cohesion of the seminiferous epithelium (BORISY and TAYLOR 1967; WALLACE 1974, ALLARD et al. 1993; CORREA et al. 2002, GIGANT et al. 2005, RUSSELL et al. 1981).

In SCCx43KO^-/-^ mice, an influence on microtubule distribution is visible. In mutant mice, alpha-tubulin immunohistochemistry shows that the entire seminiferous epithelium (with the exception of Sertoli cell nuclei and single/few spermatogonia) is immunopositive, while within wild-type tubules a stage-dependent ribbon- or cable-like structure resulting in a wheel-spoke-like staining pattern can be detected in the cytoplasm of Sertoli cells. However, it should be noted, that the seminiferous epithelium of KO testis almost entirely consists of Sertoli cells. In these tubules, denser structures reminiscent of feased ends of ropes can be detected, deflecting in all directions but not aligned with the tubular lumen. These morphological changes might be explained by the altered cell shape of Cx43-deficient Sertoli cells that are not aligned in a straight line to the lumen (as in the wild type) and are sometimes

extremely slender. In contrast, wild-type seminiferous tubules comprise a certain number of germ cells (ORTH et al. 1988), which make up a large part of the seminiferous epithelium (WING and CHRISTENSEN 1982).

Transelectron microscopy confirmed that microtubules of SCCx43KO^-/-^ mice run parallel to each other, but are not necessarily aligned with the lumen. Rather, they “follow” the altered Sertoli cell shape so that it might be assumed that the microtubules lose their orientation. Studies by Russel and Redenbach et al. have shown that microtubules can be associated with cisternae of apical endoplasmic specializations (RUSSELL 1977, REDENBACH et al. 1992) and that these “landmarks” could be important for a luminal orientation of the microtubules. Studies by Fleming et al. using an adenoviral vector for overexpression of γ-tubulin, which serves as a “starting point” for microtubule nucleation, show an accumulation of this tubulin at the contact areas of Sertoli cells with elongated spermatids (FLEMING et. al. 2003) supposing that spermatids might have an influence on where microtubule nucleation starts within the Sertoli cell and thus ultimately also on microtubule distribution/localization.

In KO-mouse models in which the coding genes for Rictor, Raptor, Cdc42 or AKAP9 were “switched off”, an absence of germ cells is also associated with altered microtubule distribution (DONG et al. 2015; VENKATESH et al. 2016; XIONG et al. 2018; SU and CHENG 2019). However, in these studies, the germ cell deficiency has different causes than in SCCx43KO^-/-^ mice so that no direct comparison of these models is possible. Further comparative studies of other knockout models leading to a loss of germ cells (e.g. SCO) or to an arrest of spermatogenesis would be very interesting in this respect.

The fact that single/few seminiferous tubules of SCCx43KO^-/-^ mice show “normal” (residual) spermatogenesis and exhibit the same staining pattern as wild-type mice when incubated with an antibody directed against α- or β-tubulin also strengthens the idea of an important influence of germ cells on microtubule distribution (and not of Cx43 protein). Using β-galactosidase immunohistochemistry, loss of Cx43 in Sertoli cells could clearly be confirmed in these seminiferous tubules (HOLLENBACH et al. 2018). However, to confirm these previous results, we performed co-staining experiments of β-galactosidase (brown) and Cx43 (red) in SCCx43KO-/- mice (Fig. E; rsp = residual spermatogenesis, LC = Leydig cells, scale bar = 20 µm). Data reveal that Sertoli cells are immunonegative for Cx43 but Sertoli cell nuclei are immunopositive for β-galactosidase. Germ cells and Leydig cells (LC) are immunonegative for β-galactosidase. Leydig cells are also immunonegative for Cx43 in KO as described by Noelke et al. (NOELKE et al. 2016).

Thus, seminiferous tubules with residual spermatogenesis can on the one side be used in comparison (1) with tubules showing a Sertoli cell only (SCO) phenotype or an arrest of spermatogenesis at the level of spermatogonia in mutant mice even in one and the same histological section, and on the other side (2) with seminiferous tubules of wild-type mice to obtain an indication of whether the observed alterations are indirect (due to the absence of germ cells) or direct (due to an interaction or binding of Cx43 with α- or β-tubulin). In the present study and the SCCx43KO mouse

model we suggest that it is not the loss of Cx43 protein but rather the absence of germ cells that to lead to the altered staining pattern of tubulins.

We thank this reviewer/you also for this comment concerning Figure 7 that a co-staining experiment with a Sertoli cell specific (nuclear) marker like Sox9 along with e.g. claudin 11 might better show that claudin 11 is diffusely distributed within the cytoplasm of the Sertoli cells.

For that purpose, and to confirm that the cytoplasm of Sertoli cells is immunopositive (and not the cytoplasm of single/few spermatogonia), we performed co-staining experiments of Sox9 and claudin 11. The blood-testis-barrier (BTB) marker claudin 11 is also known to be Sertoli cell specific. Within the normal seminiferous epithelium of wild-type mice it is located at the side of the BTB in the basal compartment of the seminiferous tubule (Fig. F; scale bar = 20 µm). In contrast, claudin 11 is diffusely distributed within the cytoplasm of Cx43-deficient Sertoli cells in SCCx43KO^-/-^ mice (see e.g. Hollenbach et al. 2018) (Fig. G; scale bar = 20 µm).

Data reveal that Sertoli cell nuclei are immunopositive for Sox9 (brown) and that the surrounding Sertoli cell cytoplasm is immunopositive for claudin 11 (red). Germ cells and Leydig cells are immunonegative (Fig. G; scale bar = 20 µm). Figure H shows a seminiferous tubule with residual spermatogenesis (rsp) and a seminiferous tubule with arrest of spermatogenesis at spermatogonial level close to each other (Fig. H; rsp = residual spermatogenesis, scale bar = 20 µm).

Additional and representative figures might be added to the final version of the manuscript depending on your and/or the editors decision.

References

ALLARD, E.,K. JOHNSON u. K. BOEKELHEIDE (1993): Colchicine disrupts the cytoskeleton of rat testis seminiferous epithelium in a stagedependent manner. Biology of Reproduction 48, 143-153

AMLANI, S. a. A. VOGL (1988): Changes in the distribution of microtubules and intermediate filaments in mammalian Sertoli cells during spermatogenesis. The Anatomical Record 220, 143-160

BATIAS C, DEFAMIE N, LABLACK A, Thepot D, Fenichel P, Segretain D, Pointis G. (1999): Modified expression of testicular gap-junction connexin 43 during normal spermatogenic cycle and in altered spermatogenesis. Cell Tissue Res. 298(1):113-21. doi: 10.1007/s004419900076. PMID: 10555545.

BORISY, G. a. E. TAYLOR (1967): The mechanism of action of colchicine. Binding of colchincine-3H to cellular protein. The Journal of Cell Biology 34, 525-533

CORREA, L., M. NAKAI, C. STRANDGAARD, R. HESS u. M. MILLER (2002): Microtubules of the mouse testis exhibit differential sensitivity to the microtubule disruptors Carbendazim and colchicine. Toxicological Sciences 69, 175-182

De Gendt K, Denolet E, Willems A, Daniels VW, Clinckemalie L, Denayer S, Wilkinson MF, Claessens F, Swinnen JV, Verhoeven G. (2011): Expression of Tubb3, a beta-tubulin isotype, is regulated by androgens in mouse and rat Sertoli cells. Biol Reprod. 85(5):934-45. doi: 10.1095/biolreprod.110.090704. Epub 2011 Jul 6. PMID: 21734264; PMCID: PMC4480425.

DONG, H., Z. CHEN, C. WANG, Z. XIONG, W. ZHAO, C. JIA, J. LIN, Y. LIN, W. YUAN, A. Z. ZHAO u. X. BAI (2015): Rictor Regulates Spermatogenesis by Controlling Sertoli Cell Cytoskeletal Organization and Cell Polarity in the Mouse Testis. Endocrinology 156, 4244-4256

FLEMING, S., P. SHANK u. K. BOEKELHEIDE (2003): gamma-Tubulin overexpression in Sertoli cells in vivo: I. Localization to sites of spermatid head attachment and alterations in Sertoli cell microtubule distribution. Biology of Reproduction 69, 310-321

GIGANT, B., C. WANG, R. RAVELLI, F. ROUSSI, M. STEINMETZ, P. CURMI, A. SOBEL u. M. KNOSSOW (2005): Structural basis for the regulation of tubulin by vinblastine. Nature 435, 519-522

HOLLENBACH, J., K. JUNG, J. NOELKE, H. GASSE, C. PFARRER, M. KOY u. R. BREHM (2018): Loss of connexin43 in murine Sertoli cells and its effect on blood-testis barrier formation and dynamics. PloS one 13, e0198100

NOELKE J, WISTUBA J, DAMM OS, FIETZ D, GERBER J, GAEHLE M, BREHM R. (2015): A Sertoli cell-specific connexin43 knockout leads to altered interstitial connexin expression and increased Leydig cell numbers. Cell Tissue Res.;361(2):633-44. doi: 10.1007/s00441-015-2126-7. Epub 2015 Feb 19. PMID: 25693895.

ORTH, J., G. GUNSALUS u. A. LAMPERTI (1988): Evidence from Sertoli cell-depleted rats indicates that spermatid number in adults depends on numbers of Sertoli cells produced during perinatal development. Endocrinology 122, 787-794

REDENBACH, D., K. BOEKELHEIDE u. A. VOGL (1992): Binding between mammalian spermatid-ectoplasmic specialization complexes and microtubules. European Journal of Cell Biology 59, 433-448

Risley M, Tan I, Roy C, Sáez J. (1992): Cell-, age- and stage-dependent distribution of connexin43 gap junctions in testes. Journal of cell science.;103 (Pt 1):81-96

RUSSELL, L. (1977): Observations on rat Sertoli ectoplasmic ('junctional') specializations in their association with germ cells of the rat testis. Tissue and Cell 9, 475-498

RUSSELL, L., J. MALONE u. D. MACCURDY (1981): Effect of the microtubule disrupting agents, colchicine and vinblastine, on seminiferous tubule structure in the rat. Tissue and Cell 13, 349-367

SU, W. u. C. CHENG (2019): Cdc42 is involved in NC1 peptide-regulated BTB dynamics through actin and microtubule cytoskeletal reorganization. FASEB journal : official publication of the Federation of American Societies for Experimental Biology 33, 14461-14478

VENKATESH, D., D. MRUK, J. HERTER, X. CULLERE, K. CHOJNACKA, C. CHENG u. T. MAYADAS (2016): AKAP9, a Regulator of Microtubule Dynamics, Contributes to Blood-Testis Barrier Function. The American Journal of Pathology 186, 270-284

WALLACE, S. (1974): Colchicine. Seminars in Arthritis and Rheumatism 3, 369-381Wenz J, Hess R. Characterization of stage-specific tyrosinated alpha-tubulin immunoperoxidase staining patterns in Sertoli cells of rat seminiferous tubules by light microscopic image analysis. Tissue & cell. 1998;30(5):492-501.

Wenz JR, Hess RA. (1998): Characterization of stage-specific tyrosinated alpha-tubulin immunoperoxidase staining patterns in Sertoli cells of rat seminiferous tubules by light microscopic image analysis. Tissue Cell.;30(5):492-501. doi: 10.1016/s0040-8166(98)80028-1. PMID: 9839471.

Wolf, K. W. a. Winking, H. (1996): The organization of microtubules and filamentous actin in cytospin preparations of Sertoli cells from *w/w* *^Wei^* mutant mice devoid of germ cells. *Anat Embryol* **193**, 413–417. https://doi.org/10.1007/BF00186698

WING, T. a. A. CHRISTENSEN (1982): Morphometric studies on rat seminiferous tubules. The American Journal of Anatomy 165, 13-25

XIONG, Z., C. WANG, Z. WANG, H. DAI, Q. SONG, Z. ZOU, B. XIAO, A. Z. ZHAO, X. BAI u. Z. CHEN (2018): Raptor directs Sertoli cell cytoskeletal organization and polarity in the mouse testis. Biology of Reproduction 99, 1289-1302

Reviewer 2:

Thank you for pointing out that the text still contains grammatical errors. We have had the manuscript checked by a native speaker and corrected the errors.
